# Letter to the Editor: Reconsidering the Leap from Descriptive Association to Causal Safety in the Beginnings Study

**DOI:** 10.1016/j.advnut.2026.100711

**Published:** 2026-09-04

**Authors:** Chunsong Pang, Lifen Zhao

**Affiliations:** Department of Laboratory Medicine, Second Hospital of Dalian Medical University, Dalian, China

Dear Editor:

We read with interest the comprehensive narrative synthesis by da Cruz et al. [1] entitled “Longitudinal evaluation from birth to adolescence of soy-protein based infant formula compared to cow-milk based formula and breastfeeding: A comprehensive summary of findings.” The study tracks 600 infants from 3 mo to 14 y across growth, body composition, cardiovascular, neurodevelopmental, and reproductive outcomes. It confirms that human milk (HM) is consistently linked to lower adolescent adiposity, while soy-based (SF) and cow milk–based (MF) formulas show broadly similar results across most domains—a useful descriptive finding for clinicians and guideline developers. However, the leap from “no statistically significant long-term differences between SF and MF” to claims of causal safety—and implicitly, that “modern SF is safe under appropriate clinical guidance”—exceeds what the observational design and methodological limitations support.

A key concern is whether the apparent similarity between SF and MF reflects true biological equivalence—or simply results from selective attrition. Of the original 600 infants, only 190 (31.7%) completed the 14-y visit, with uneven retention across groups (HM: 76; MF: 62; SF: 52). Because loss to follow-up is linked to both feeding group (e.g., HM families often differ in resources and engagement) and outcomes (adiposity, cardiometabolic risk, pubertal development), complete-case analysis may mask real differences—the “healthy completer” effect. Without sensitivity analyses—such as inverse probability weighting or pattern-mixture modeling—to address this bias, the “no difference” finding is empirically fragile [2]. And without attrition diagnostics stratified by feeding group, the 14-y results cannot be confidently generalized.

A second issue is whether the equivalence of SF and MF can be interpreted as causal safety given the observational, parent-selected design. The authors employ directed acyclic graph (DAG)-informed covariate adjustment, which is commendable. Yet, the study’s own recurrent finding—that HM is linked to persistently lower BMI and fat mass index through adolescence—confirms that feeding mode is entangled with a constellation of parental behaviors, resources, and care patterns that no adjustment set can fully neutralize [3]. If unmeasured factors influenced both why a family chose SF compared with MF (e.g., prior allergy history, gastrointestinal symptoms, clinician advice, or cultural dietary orientation) and long-term outcomes, then SF ≈ MF after adjustment is necessary but not sufficient evidence that SF is inherently neutral. It is an association equally compatible with “no meaningful harm” and with “harm or benefit exists but is masked by selection.” In nonrandomized designs, absence of evidence should be reported with its precision—confidence intervals and statistical power—rather than framed as evidence of absence.

A related concern is whether the wide range of endpoints truly supports a safety conclusion. The study assesses many measures—anthropometry, dual-energy X-ray absorptiometry (DXA)/peripheral quantitative computed tomography (pQCT), heart rate variability, microbiota diversity and composition, urinary metabolomics, electroencephalogram band power, temperament, and pubertal sonography—most as exploratory. Yet, the conclusions state that modern SF is safe and call for policy changes. Two issues undermine this leap. First, with so many comparisons, isolated “significant” findings (e.g., in autonomic function or specific microbes) risk false positives without multiplicity correction. Second, 14 years is too short to assess true long-term safety: outcomes like fertility, menstrual regularity, and adult metabolic phenotypes emerge later and remain unmeasured. Findings reassuring up to age 14 are valid; claiming they confirm long-term safety is not [4].

Finally, the clinical translation of “no observed adverse effects” demands caution. The authors conclude that modern SF is safe under clinical guidance, citing absent differences in reproductive organ size or pubertal timing. However, safety is not merely the absence of overt harm at a single time point; it also encompasses whether the product is needed for a given infant and whether alternatives exist. Current guidelines reserve SF primarily for confirmed cow milk allergy when extensively hydrolyzed formulas are unsuitable. The estrogenic activity of soy isoflavones—although not conclusively harmful in humans—remains biologically plausible for vulnerable subgroups, including premature infants and those with thyroid disorders [5]. Moreover, the study’s own data show that SF-fed adolescents had intermediate fat mass index and waist circumference between HM and MF—a pattern that, although not statistically significant, aligns with concerns that early diet may subtly influence long-term metabolic set points.

Taken together, da Cruz et al. [1] present a landmark descriptive study of soy formula outcomes. Although SF and MF show similar results across most measures, this does not prove safety. Attrition, observational design, multiple comparisons, and the 14-y limit preclude causal inference. Association should not be mistaken for clinical actionability. SF should remain restricted to infants with confirmed cow milk allergy who cannot tolerate hydrolyzed formula.

## Author contributions

Both authors read and approved the final manuscript

## Declaration of Generative AI and AI-assisted technologies in the writing process

The author(s) declare that no generative AI or AI-assisted technologies were used in the writing of this manuscript.

## Funding

The authors reported no funding received for this study.

## Conflict of interest

The authors report no conflicts of interest.
